# Basi‐parallel anatomical scanning–magnetic resonance imaging for the diagnosis of reversible cerebral vasoconstriction syndrome of the basilar artery: a case report

**DOI:** 10.1002/ams2.300

**Published:** 2017-07-28

**Authors:** Akitaka Yamamoto, Yukinari Omori, Akihiro Shindo, Hiroshi Imai, Hidenori Suzuki

**Affiliations:** ^1^ Emergency and Critical Care Center Mie General Medical Center Yokkaichi Mie Japan; ^2^ Department of Neurosurgery Mie University Graduate School of Medicine Tsu Mie Japan; ^3^ Emergency and Critical Care Center Mie University Hospital Tsu Mie Japan; ^4^ Department of Neurology Mie University Graduate School of Medicine Tsu Mie Japan

**Keywords:** Calcium channel blockers, cerebral vasospasm, cerebrovascular diseases, magnetic resonance angiography, thunderclap headache

## Abstract

**Case:**

Reversible cerebral vasoconstriction syndrome (RCVS) is an increasingly recognized and important cause of thunderclap headache. Delays in diagnosis may cause deterioration of symptoms and concomitant unnecessary investigations. However, the diagnosis of RCVS in the acute stage remains difficult.

A 41‐year‐old man was admitted to the emergency department for severe, recurrent occipital headaches. The results of initial computed tomography and magnetic resonance imaging (MRI) of the brain and cerebrospinal fluid analysis were normal. Magnetic resonance angiography (MRA) showed multisegmental luminal stenosis of the basilar artery. Basi‐parallel anatomical scanning (BPAS)‐MRI, illustrating the outer contour of the vertebrobasilar artery, also showed multisegmental stenosis.

**Outcome:**

The patient was speculated as having RCVS and was treated with oral lomerizine hydrochloride. Repeat MRA and BPAS‐MRI 2 months later showed resolution, confirming RCVS.

**Conclusion:**

Combined with MRA, BPAS‐MRI is an effective and non‐invasive imaging method for diagnosis of RCVS of the basilar artery.

## Introduction

Headache is one of the most common primary complaints presenting to the emergency department (ED). A thunderclap headache (TCH), is a hyperacute and severe headache that reaches maximum intensity within 1 min. Differential diagnosis of TCH is broad and includes intracranial aneurysm, subarachnoid hemorrhage (SAH), cerebral venous thrombosis, caudocervical artery dissection, ischemic stroke, pituitary apoplexy, and intracranial infection.^1^ Another cause of TCH that has gained increased recognition in recent years is reversible cerebral vasoconstriction syndrome (RCVS). At present, the diagnosis of RCVS can only be confirmed by follow‐up imaging showing resolution of blood vessel irregularities. Therefore, it is difficult to diagnose RCVS in the acute stage using initial neuroimaging, such as computed tomography (CT) and magnetic resonance imaging (MRI).

Here, we report the diagnosis and temporal course of RCVS by observing the outer appearance of the intracranial basilar artery in basi‐parallel anatomical scanning (BPAS) and magnetic resonance angiography (MRA).

## Case

A 41‐year‐old man, non‐smoker, taking no medications, and having no history of headache, presented with a first experience of a sudden‐onset and severe occipital headache that reached peak intensity within 1 min after bending his neck downward. There was no associated photophobia, neck stiffness, fever, visual loss, limb weakness, convulsion, or loss of consciousness. Treatment with loxoprofen sodium hydrate relieved the headache after 4 h. However, the headache recurred the next day and persisted for 6 days despite daily loxoprofen sodium hydrate; therefore, he visited our ED.

**Figure 1:**
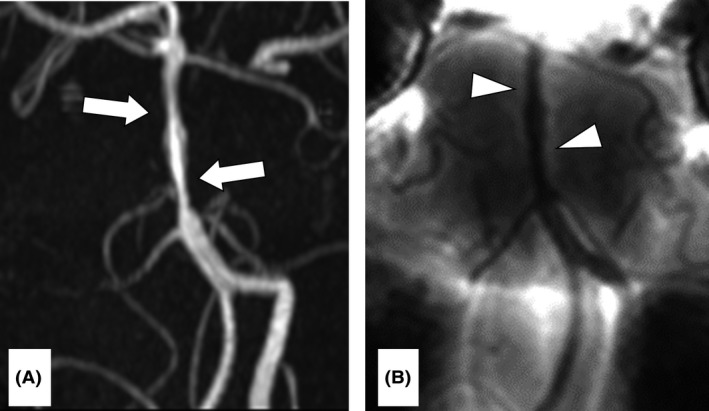
Initial imaging in the emergency department of a 41‐year‐old man with reversible cerebral vasoconstriction syndrome of the basilar artery. A, Magnetic resonance angiography of the brain showing multifocal segmental narrowing of the basilar artery (arrows). B, Basi‐parallel anatomical scanning–magnetic resonance imaging showing a multifocal narrowing vascular lesion with the same appearance as on magnetic resonance angiography.

In the ED, the patient was afebrile with a pulse of 63 b.p.m. and blood pressure of 112/66 mmHg. The results of a neurological examination, including mental status, and those of a laboratory examination, including C‐reactive protein levels, were normal. Although brain CT and MRI were normal, MRA showed multisegmental narrowing of the basilar artery (Fig. 1A), which was in agreement with the findings of BPAS‐MRI (Fig. 1B). Cerebrospinal fluid (CSF) investigation indicated no inflammation or infection.

**Figure 2:**
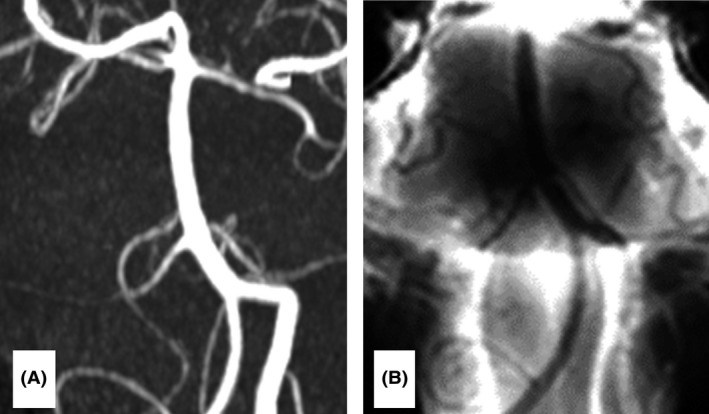
Repeat magnetic resonance angiography (A) and basi‐parallel anatomical scanning–magnetic resonance imaging (B) showing complete resolution of the vasoconstriction in a 41‐year‐old man with reversible cerebral vasoconstriction syndrome of the basilar artery.

Based on these findings, a diagnosis of RCVS was presumed, and thus, treatment with the calcium channel blocker lomerizine hydrochloride (10 mg/day) was started. Repeat MRA and BPAS‐MRI on post‐admission day 7 showed mild resolution of vasoconstriction and no hyperintense lesion on a diffusion‐weighted image. On post‐admission day 8, the symptoms had resolved and the patient was discharged uneventfully. A follow‐up brain MRI 2 months after the initial symptoms showed complete resolution of the segmental narrowing on MRA (Fig. 2A) and BPAS‐MRI (Fig. 2B), which is consistent with a diagnosis of RCVS.

## Discussion

We described the findings and diagnosis of RCVS of the basilar artery using BPAS‐MRI, which is a recently developed imaging method to evaluate the appearance of the outer vessels. Reversible cerebral vasoconstriction syndrome is characterized by acute severe headache, often like TCH with or without additional neurologic symptoms, and radiographic findings of diffuse segmental cerebral vasoconstriction of the intracranial internal carotid arteries, basilar artery, and other major arteries of the circle of Willis that are reversible in weeks to months.^2^, ^3^

Imaging techniques used for the assessment of RCVS include digital subtraction angiography (DSA), CT angiography, MRA, and transcranial Doppler ultrasonography.^4^ Digital subtraction angiography has widely been applied as a gold standard for the diagnosis of RCVS. However, this method is invasive and impractical for frequent follow‐up, which may be required to document the reversal of vasoconstriction. Although CT angiography can also be used to assess the intracerebral vessels if MRI is not readily available, radiation exposure limits its use.^5^ Magnetic resonance angiography has been confirmed as valid to evaluate vasoconstriction in patients with RCVS.^6^ However, MRA is potentially affected by flow‐related inhomogeneities. Transcranial Doppler ultrasonography is an inexpensive, non‐invasive imaging technique to monitor vasospasms of larger vessels but is subject to significant interoperator variability.^5^

Similar clinical–angiographic patterns (sudden severe headache and diffuse vasospasm) have been described in patients with SAH and primary central nervous system vasculitis. In this case, because of normal findings on initial brain CT scan and normal CSF examination, a diagnosis of SAH was excluded. The abrupt onset of headaches, benign CSF examination findings, and complete clinical and angiographic resolution without immunosuppressive treatment excluded the possibility of primary central nervous system vasculitis.

An important differential diagnosis of RCVS is cerebral artery dissection, a potentially fatal disease with specific findings on neuroimaging, which also shows segmental narrowing and dilatation (string of beads) of one or more cerebral arteries. Concern for vascular dissection in young adults complaining of occipital headache may be an indication for urgent MRI. Establishment of a diagnosis is important because of differences in further management between dissection and RCVS. An MRI technique developed by Nagahata *et al*.,^7^ BPAS‐MRI requires only a 2‐cm‐thick, heavily T2‐weighted coronal image that is parallel to the clivus. It has been used to achieve accurate 3‐D anatomic views of the vertebrobasilar arteries. Imaging with BPAS demonstrates the outer contour of the vertebrobasilar artery without the influence of flow or thrombus, whereas other established imaging techniques, such as MRA and DSA, show the inner contours of the vessels. In dissection, the intravascular lumen (shown by conventional MRA) is narrow and the surface of the vessel (shown by BPAS) is extended.^8^ Herein, the lumen and surface of the basilar artery were similarly described; thus, a diagnosis of RCVS was considered.

Treatment with a calcium channel blocker, such as lomerizine hydrochloride, a highly selective vasodilator of the cerebral blood vessels,^9^ for 4–8 weeks is considered acceptable for RCVS. As this regimen is based only on expert opinion and few case series,^1^ further studies will be needed to evaluate the efficacy of lomerizine hydrochloride for treating RCVS.

## Conclusion

Reversible cerebral vasoconstriction syndrome should be considered in the differential diagnosis of TCH. Early and accurate diagnosis is of utmost importance for appropriate management and prognosis. The approach for patient evaluation should be to rule out other causes of RCVS, such as SAH, primary central nervous system vasculitis, and dissection, which overlap considerably with RCVS. Although its application is limited to the vertebrobasilar system, BPAS‐MRI could have a supplementary role to MRA in the diagnosis of RCVS of the basilar artery.

## Disclosure

Informed consent for the publication of this case report and any accompanying images was obtained from the patient. Conflicts of Interest: Authors have no conflict of interest.
